# Establishing Quality Control Metrics for Large-Scale Plasma Proteomic Sample Preparation

**DOI:** 10.1021/acsmeasuresciau.3c00070

**Published:** 2024-04-29

**Authors:** Nekesa C. Oliver, Min Ji Choi, Albert B. Arul, Marsalas D. Whitaker, Renã A. S. Robinson

**Affiliations:** † Department of Chemistry, Vanderbilt University, Nashville, Tennessee 37235, United States; ‡ Vanderbilt Memory and Alzheimer’s Center, Vanderbilt University Medical Center, Nashville, Tennessee 37212, United States; § Vanderbilt Institute of Chemical Biology, Vanderbilt University, Nashville, Tennessee 37232, United States; ∥ Vanderbilt Brain Institute, Vanderbilt University, Nashville, Tennessee 37232, United States; ⊥ Department of Neurology, Vanderbilt University Medical Center, Nashville, Tennessee 37232, United States

**Keywords:** quality control, plasma, proteins, automation, mass spectrometry, sample preparation, large cohort

## Abstract

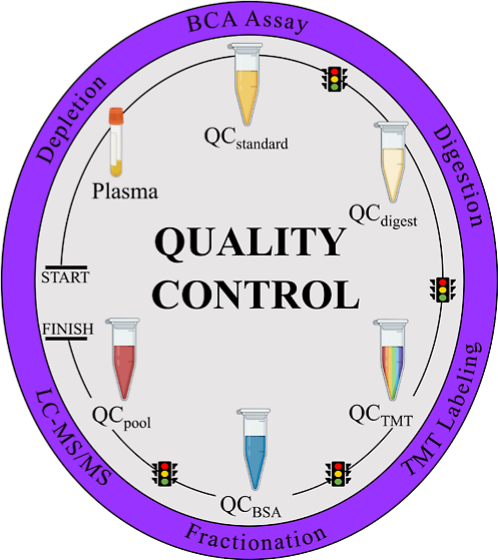

Large-scale plasma proteomics studies have been transformed due to the multiplexing and automation of sample preparation workflows. However, these workflows can suffer from reproducibility issues, a lack of standardized quality control (QC) metrics, and the assessment of variation before liquid chromatography–tandem mass spectrometry (LC–MS/MS) analysis. The incorporation of robust QC metrics in sample preparation workflows ensures better reproducibility, lower assay variation, and better-informed decisions for troubleshooting. Our laboratory conducted a plasma proteomics study of a cohort of patient samples (*N* = 808) using tandem mass tag (TMT) 16-plex batches (*N* = 58). The proteomic workflow consisted of protein depletion, protein digestion, TMT labeling, and fractionation. Five QC sample types (QC_std_, QC_dig_, QC_pool_, QC_TMT_, and QC_BSA_) were created to measure the performance of sample preparation prior to the final LC–MS/MS analysis. We measured <10% CV for individual sample preparation steps in the proteomic workflow based on data from various QC sample steps. The establishment of robust measures for QC of sample preparation steps allowed for greater confidence in prepared samples for subsequent LC–MS/MS analysis. This study also provides recommendations for standardized QC metrics that can assist with future large-scale cohort sample preparation workflows.

## Introduction

Protein discovery experiments to identify potential biomarker candidates rely on the analysis of a large number of clinical samples.^1,2^ These large-scale (*N* > 100) proteomic studies have the potential to reveal insights into the underlying biochemical mechanisms of various diseases such as cancer,^1^ sepsis,^2,3^ hypertension,^4,5,^ and Alzheimer’s disease.^6^ Quantitative liquid chromatography–tandem mass spectrometry (LC–MS/MS) approaches, in addition to immunoaffinity proximity extension assays (Olink)^7^ and aptamer-based assays (SomaScan),^8−10^ allow researchers to measure relative protein levels in patient samples.^7−10^ LC–MS/MS-based proteomics is a complementary antibody-based assay when profiling a patient’s clinical disease status.^11,12^ LC–MS/MS methodologies can provide enhanced specificity, cost-effectiveness, and the ability to manage sample complexity and quantify thousands of proteins in a single MS run.^13^ Biological samples, such as brain tissue,^14,15^ single-cells,^16,17^ or blood plasma,^18,19^ are of particular interest, especially in the context of cardiovascular and neurodegenerative diseases. Plasma, which is often collected during routine clinical visits, remains a useful proxy of disease.^20^

Prior to LC–MS/MS analysis, sample preparation is critical. Plasma sample preparation steps can be tailored for the desired discovery or targeted proteomic analyses. For discovery-based LC–MS/MS analysis, large cohorts of plasma samples are prepared with an extensive proteomic sample preparation workflow that can include (1) protein depletion, (2) protein digestion, (3) isotopic or isobaric labeling,^21,22^ and (4) peptide fractionation.^23^ Ideally, sample preparation workflows should be designed to minimize sample handling and operator-generated biases. However, sample preparation can be tedious, error-prone, and susceptible to reproducibility issues.^24−27^ Automation with robotic liquid handlers relieves the burden of sample preparation while also reducing variability between operators and samples.^28^ These handlers are also embraced for reducing the cost of routine biomarker discovery and development.^29^ Automation of sample preparation facilitates increased throughput and reproducible quantitation of biomarker candidates;^30^ however, this has been limited to individual steps of the workflow.^13,21,31^ Additionally, quality control (QC) efforts have been geared toward tracking variations in proteomic workflows but have focused on QC within MS acquisition.^32,33^

QC for large-scale proteomics is necessary to track the variation between sample batches and workflow steps.^34^ The introduction of internal and external QC sample types can aid in the assessment of laboratory performance and help promote high-quality data.^35^ Internal controls can be added as a QC check of multiple sample preparation steps, LC–MS acquisition,^36,37^ and data normalization. External controls are added as interbatch checks that are used to assess instrument performance.^38,39^ Together, internal and external QC metrics strengthen confidence in the LC–MS/MS analysis and study data, in addition to enabling other researchers to reproduce and build upon published studies.^21^

This study sought to establish QC metrics for sample preparation steps toward the analysis of a large cohort (*N* = 808) using an automated, high-throughput plasma proteomic workflow. Previously, our laboratory established analytical metrics to assess QC in LC–MS/MS^40^ and LC–MS^3^ data acquisition for tandem mass tag (TMT)-based proteomics analysis.^39^ Those metrics easily allowed operators to determine when a sample batch was out-of-specification (OOS) and enabled real-time troubleshooting. Here, we created five QC sample types (QC_std_, QC_dig_, QC_pool_, QC_TMT_, and QC_BSA_) and used them strategically to (1) measure instrument performance, (2) check the efficiency of the individual workflow steps, and (3) guide operator actions that progressed the sample preparation workflow.

## Materials and Methods

### Sample Selection

Plasma samples (*N* = 808) from African American/Black normotensive (*N* = 404) and hypertensive (*N* = 404) adults (age 45+ years) were obtained from the Southern Community Cohort Study (SCCS) (https://www.southerncommunitystudy.org/about-the-sccs.html), a case-control study established to elucidate and discover sources of higher rates of various diseases among African American adults in the Southern United States.^41^ Informed consent and baseline health questionnaires were obtained from participants, and this secondary analysis of all data was performed in accordance with the Vanderbilt University Institutional Review Board (IRB). A full proteomic analysis of this SCCS participant subset will be presented elsewhere. Here, the samples were used to generate and assess QC metrics for the sample preparation steps, as described below.

### Plasma Depletion

Human plasma standard (Sigma) aliquots (*N* = 116) were created by reconstituting the dry standard (5 mL) in LC–MS water (1:1) and storing them at −30 °C until ready for use. Aliquots (40 μL) were prepared, in duplicate, for depletion through a 1:4 plasma/buffer dilution using buffer A of a proprietary and ready-to-use, dual buffer system (Agilent, Santa Clara).^42^ Diluted samples were centrifuged at 16,000*g* for 1 min at 4 °C through a 0.22 μM filter (MilliporeSigma, Burlington). Sample flow-throughs (130 μL) were injected onto a Multiple Affinity Removal Human 14 (MARS-14) depletion column (4.6 × 100 mm, Agilent, Santa Clara) installed on a Waters e2695 high-performance liquid chromatography (HPLC) system with a fraction manager analytical (FMA) module and a 2998 photodiode array (PDA) detector. The unbound fraction was collected in a 5 mL glass vial (Thermo Fisher Scientific, Waltham), combined with the duplicate unbound fraction, and concentrated by using 10 kDa centrifugal filters (Amicon, Burlington). Concentrates of depleted human plasma standard (QC_std_) were quantified by bicinchoninic acid (BCA) protein assays (Thermo Fisher Scientific, Waltham). To monitor daily HPLC performance, QC_std_ samples were used to conduct retention time (*t*_R_) peak analysis and MARS-14 column efficiency checks before being combined into a bulk stock and stored at −80 °C until further analysis.

### Automated Protein Digestion

Depleted samples (100 μg) were assigned into batches, each consisting of 14 SCCS participants and two QC_std_ samples. Using a robotic liquid handler (Biomek i7 Automated Workstation) (Beckman Coulter, Brea), batches were arranged onto 96-well plates (i.e., ten plates total for the study) that each held 12 randomized QC_std_ samples. Robotic liquid handler methods were designed such that eight plates were processed over two days and the remaining two on the third day. All reagents were made fresh daily. Batches were adjusted by volume in 1 M ammonium bicarbonate (Honeywell, Charlotte). Proteins were reduced with 200 mM dithiothreitol (DTT) (Thermo Fisher Scientific, Waltham) for a 45 min incubation at 55 °C, alkylated in 200 mM iodoacetamide (IAM, 98%) for a 30 min dark incubation at 25 °C, and digested using trypsin/Lys-C (Promega, Madison) at a 1:50 enzyme/substrate ratio for 14 h at 37 °C. Digested samples were acidified with 5% formic acid (Thermo Fisher Scientific, Waltham), and the digested QC_std_ (QC_dig_) wells were confirmed with a pH strip test (pH ≤ 3). Here, the robotic liquid handler switched to an automated cleanup step using BioPureSPE C_18_ 96-well plates (The Nest Group, Ipswich) and the Positive Pressure X-Well SPE Extractor (PPA) (Ultimaration, Rostock) accessory. Sample volumes were reduced by evaporation to ensure that the volume could be transferred in a single step from sample plates to BioPureSPE plates. Six QC_dig_ samples from each sample plate were manually combined into low-binding Eppendorf tubes and designated for TMT labeling. The remaining six underwent digestion checks using the LC–MS methods described below. Plates and designated QC_dig_ samples were dried overnight, sealed, and stored at −80 °C until ready for further analysis.

### Automated Sample Pooling and TMT Labeling

On the Biomek and prior to TMT labeling, SCCS participant samples were reconstituted in 100 mM tetraethylammonium bromide (TEAB, pH 8.5) (MilliporeSigma, Burlington), and a pooled-plasma stock solution (*S*_pool_) was prepared by combining an equimolar amount of peptide from each sample. Samples (25 μg) were then arranged onto new 96-well plates such that each contained five batches, 4 wells of *S*_pool_, and 8 wells of QC_dig_. Samples and *S*_pool_ wells were labeled with TMTpro 16-plex and TMTzero (TMT^0^), respectively. Simultaneously, in the second-to-last column of a sample processing plate, QC_dig_ was also labeled with TMTpro in an 8-plex fashion that spanned across two plates. Individual TMT tags were reconstituted with anhydrous acetonitrile to a 1:25 reagent/sample ratio and added to their respective wells. Plates were sealed and incubated at room temperature for 1 h before 5% hydroxylamine was added, and the reaction was quenched after 15 min. QC_dig_ became QC_TMT_ after successful labeling. All batches were pooled accordingly, and TMTzero-labeled QC_pool_ samples were combined into a bulk stock. Samples were desalted, dried, and stored at −80 °C before further analysis. QC_TMT_ underwent LC–MS/MS analysis to confirm the labeling efficiency (LE).

### Reversed-Phase Fractionation

Bovine serum albumin (BSA, 1 mg) (Sigma-Aldrich, Milwaukee) was manually digested to serve as a QC sample to conduct HPLC performance monitoring. BSA was diluted in 50 mM Tris with 8 M urea (1 μg/μL), reduced in 25 mM DTT (1:40 protein/reagent), and incubated in water at 37 °C for 30 min. IAA (1:80 protein/reagent) was added for alkylation before a 30 min incubation on ice in the dark. l-Cysteine (25 mM) was added before a 30 min incubation on a shaker at room temperature to quench the reaction. The sample was diluted 10-fold with 20 mM Tris and 10 mM CaCl_2_. Trypsin in 50 mM acetic acid was added at a 1:50 enzyme/substrate molar ratio before incubation in water at 37 °C for 14 h. BSA peptides were acidified with formic acid (pH = 3), desalted with HLB filters, and dried. Digested BSA (QC_BSA_) and multiplexed samples were reconstituted to 1 μg/μL in 1.0 mM ammonium formate and 2% acetonitrile (pH = 10). QC_BSA_ and samples, 100 and 300, μL respectively, were injected onto a Zorbax Extend300C_18_ analytical column (4.6 mm i.d. × 12.5 mm, 5 μm) with a matching guard column (Agilent, Santa Clara) on a Waters HPLC e2695 with PDA and FMA modules. Over the 60 min gradient, injections were separated using high-pH (pH = 10) reversed-phase fractionation with 4.5 mM ammonium formate and 2% acetonitrile as Buffer A and 4.5 mM ammonium formate and 90% acetonitrile as Buffer B. Gradient conditions were as follows: 0–10 min, 0% B; 10–41 min, 10–30% B; 41–45 min, 30–60% B; 45–46 min, 60–90% B; 46–49 min, 10% B; 49–50 min, 10–90% B; 50–60 min, 90% B. One fraction was collected each minute, for a total of 96 fractions that were concatenated into a final set of 24. Daily, two SCCS batches were processed and dried by centrifugal evaporation.

### LC–MS/MS Analysis

Digestion efficiency checks were conducted using an Ultimate 3000 nano ultrahigh-performance liquid chromatography system coupled with a Q-Exactive HF mass spectrometer (Thermo Fisher Scientific, Waltham) operated in positive electrospray ionization mode. The QC_dig_ and QC_pool_ samples were reconstituted to a 0.25 μg/μL concentration in 0.1% formic acid and loaded onto a C_18_ trap column (75 μm i.d. × 2 cm, 100 Å, 3 μm; Thermo Fisher Scientific, Waltham) at 2 μL/min using a 2% acetonitrile and 0.1% formic acid loading buffer. Peptides were separated at 0.3 μL on an in-house XBridge BEH C_18_, 2.5 μm packed analytical column (100 μm i.d. × 28 cm) (Waters Corporation, Milford) using solvent A (water with 0.1% formic acid) and solvent B (acetonitrile with 0.1% formic acid). QC samples were separated on the following LC gradient: 0–93 min, 10% B; 93–134 min, 18% B; 134–139 min, 27% B; 139–150 min, 85% B; 150–160 min, 10% B. Full MS spectra were acquired in the Orbitrap (400–1600 *m*/*z*, 120,000 resolutions, microscans = 1). The automatic gain control (AGC) target was set to 5.0 × 10^5^ with a maximum injection time set to 86 ms.

For TMT efficiency checks, data-dependent acquisition mode was used to acquire the top 15 MS/MS spectra using an Orbitrap Fusion Lumos Tribrid system (Thermo Fisher Scientific, Waltham) in positive nanospray ionization mode. All samples were analyzed in duplicate with the following LC gradient: 0–14 min, 2% B; 14–17 min, 2–7% B; 17–100 min, 7–16% B; 100–155 min, 16–25% B; 155–160 min, 25–85% B; 160–168 min, 85% B; 168–170 min, 85–4% B; and 170–180 min, 4% B. Full MS scans were collected using a 300 °C ion transfer tube temperature and Orbitrap isolation over a *m*/*z* range of 400–1600. The maximum injection time was 86 ms, with a resolution of 120,000 and microscans set to 1. Monoisotopic peptide peak determination included charge states from 2 to 6. Tandem spectra were acquired using a quadrupole isolation window of 0.7 *m*/*z* and a normalized collision energy of 35%. Peptide fragmentation was performed in the high-energy C-trap with the normalized AGC target set to 250%.

### Data Analysis

.RAW files were analyzed using the Proteome Discoverer (PD) software (v 2.5) and searched against the Uniprot reviewed human protein database (04/02/2021, 79,740 sequences). The following SEQUEST-HT parameters were used in this database search: dynamic modifications of methionine oxidation (15.995 Da), TMTpro on lysine residues (224.152 Da), and peptide N-termini (224.152 Da); a maximum of two missed cleavage sites; peptide lengths ranging 6–144; precursor mass tolerance of 10 ppm; and a fragment mass tolerance of 0.02 Da. Peptides and their corresponding proteins were filtered to only include those identified with a false discovery rate (FDR) of <1% and those that had at least two peptide spectral matches (PSMs). Protein and peptide abundances were determined using the precursor ion quantifier node for QC_dig_, whereas the reporter ion node was used for TMTpro-labeled QC_TMT_.

### Quality Control Metrics

**Figure 1:**
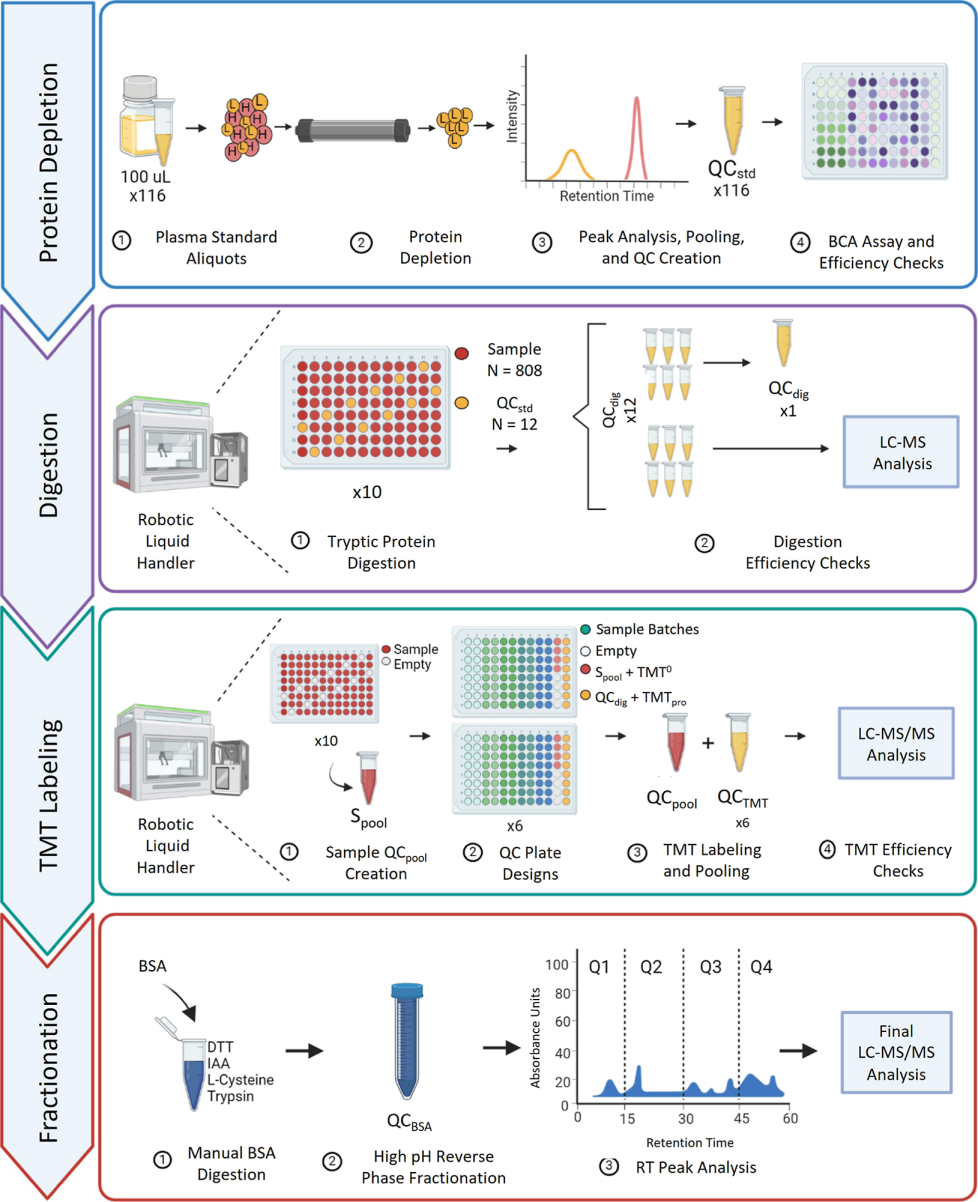
Automated, high-throughput plasma proteomic workflow with integrated QC metrics. Five different QC sample types were created for the sample preparation of a large cohort (SCCS, *N* = 808) to measure instrument performance, check efficiency, and progress the workflow. QC samples were processed simultaneously with the cohort and included (1) a bulk QC_std_ sample for protein depletion, created from combining 116 human plasma standard samples (20 μL each) and used to check depletion efficiency; (2) a stock QC_dig_ sample for tryptic digestion, created from combining six of the 12 wells per plate of digested QC_std_ and used for digestion efficiency checks; (3) a bulk QC_pool_ for final LC–MS analysis and created from a TMTzero-labeled equimolar combination of all digested participant samples (*S*_pool_); (4) six QC_TMT_ samples that are 16-plex batches of TMTpro-labeled QC_dig_ to check LE; and (5) forty QC_BSA_ aliquots to track HPLC performance during high-pH (pH = 10) fractionation. Each workflow step includes an efficiency check and supports the reproducibility of sample preparation, leading to the final LC–MS/MS analysis. Created with BioRender.com. Abbreviations: BSA = bovine serum albumin, MARS-14 = multiple affinity removal system top 14.

In addition to the five QC sample types created throughout this workflow, LC–MS/MS-based QC checks were performed after the automated protein digestion and TMT labeling steps. For digestion, operators utilized the Freestyle software (version 1.8 SP1) to visually analyze full MS spectra and confirm the presence of peptides in QC_dig_ samples. Upon confirmation, peptide and protein identifications were determined with PD. A digestion efficiency specification of >80% of identified peptides having no missed cleavages was evaluated before samples progressed for QC_pool_ creation. For TMT labeling, LC–MS/MS checks were performed to confirm the presence of the TMT tags at their respective *m*/*z* ratios. Operators visually assessed MS/MS spectra for the 16 TMTpro tags. A ≥98% average LE specification was selected based on the manufacturer’s recommendations, the results herein, and in recent quantitative proteomic studies.^22,43,44^ For fractionation, BSA was selected as the daily QC sample type, as it produced a less complex chromatogram and allowed operators to evaluate retention time (*t*_R_) reproducibility. Quadrants were selected based on the length of the fractionation method. The two peaks with the highest abundance within each quadrant were selected for daily *t*_R_ tracking. If multiplexed or QC samples produced peaks that fell outside the *t*_R_ ranges, the separation was considered OOS. These alerted operators to reanalyze, repeat, or further troubleshoot to ensure the quality preparation of samples. This provided a robust pass/fail QC system that progressed samples to the next step of the workflow (Figure 1).

## Results and Discussion

A summary of the automated plasma proteomics sample preparation workflow (Figure 1) details the QC sample types and metrics created in this discovery study. This workflow included five QC sample types (QC_std_, QC_dig_, QC_pool_, QC_TMT_, and QC_BSA_) to coincide with the major steps of the preparation workflow (Table 1). A bulk QC_std_ was generated by combining MARS-14 depletions (*N* = 116) of a human plasma standard that passed BCA assays and protein depletion efficiency checks. To automate protein digestion, 96-well plates (*N* = 10) were designed with QC_std_ sample wells (*N* = 12) arranged in every column across each plate. QC_std_ became QC_dig_ upon protein digestion with trypsin/Lys-C. Half of the QC_dig_ wells per plate were combined into a bulk stock. The remaining QC_dig_ samples were used for efficiency checks by using LC–MS/MS analysis. *S*_pool_ was generated prior to the TMT labeling of patient sample batches and QC_dig_ samples. Well plates (*N* = 12) included a column with four wells of *S*_pool_ and another with eight wells of QC_dig_. *S*_pool_ became QC_pool_ upon TMT^0^ labeling, while QC_dig_ became QC_TMT_ upon TMTpro (16-plex) labeling. QC_pool_ was reserved for final LC–MS/MS analysis, while QC_TMT_ samples were pooled into their respective batches (*N* = 6) and underwent LC–MS/MS analysis for a LE check. Finally, bulk QC_BSA_ was generated from the manual digestion of BSA and used to evaluate high-pH reversed-phase peptide fractionation. Fractionation chromatograms were divided into four equal time quadrants (*N* = 4) and underwent a retention time peak analysis. Overall, this sample preparation workflow was strategically designed and planned to embed multiple layers of QC.

**Table 1:** Quality Control Sample Types

name	description	specification
QC_std_	protein depleted and concentrated human plasma standard	≥90% MARS-14 depletion efficiency
QC_dig_	tryptic digest of QC_std_	≥80% proteins with no missed cleavages
QC_pool_	equimolar combination of all plasma sample peptides	a
QC_TMT_	TMT-labeled QC_dig_	≥98% TMTpro labeling efficiency
QC_BSA_	tryptic digest of bovine serum albumin	within the retention time quadrant of the gradient

a Reserved for final LC–MS/MS analyses.

### MARS-14 Depletion Efficiency

Commercially available human plasma standard aliquots were depleted (QC_std_) and used to track the MARS-14 column performance. For protein depletion on the MARS-14 column, the manufacturer reports, efficiencies up to 99% should be attainable.^45^ Our laboratory has previously determined that 90% column recovery is possible based on smaller cohorts and QC_std_ analyses.^46^ QC_std_ samples (*N* = 116) were acquired, and retention times of unbound fractions were highly reproducible with a 2.90% coefficient of variation (CV) (Table 2). Unbound fractions were concentrated, and protein concentration was determined with BCA.

**Table 2:** Peak Analysis of QC_std_^a^

	RT	peak area	height
average	11.74	7.49 × 10^7^	6.63 × 10^5^
SD	0.34	2.79 × 10^7^	7.69 × 10^4^
% CV	2.90	37.29	11.60

a Depleted human plasma standard aliquots (*N* = 116) are denoted as QC_std_; abbreviations: CV, coefficient of variance; SD, standard deviation.

**Figure 2:**
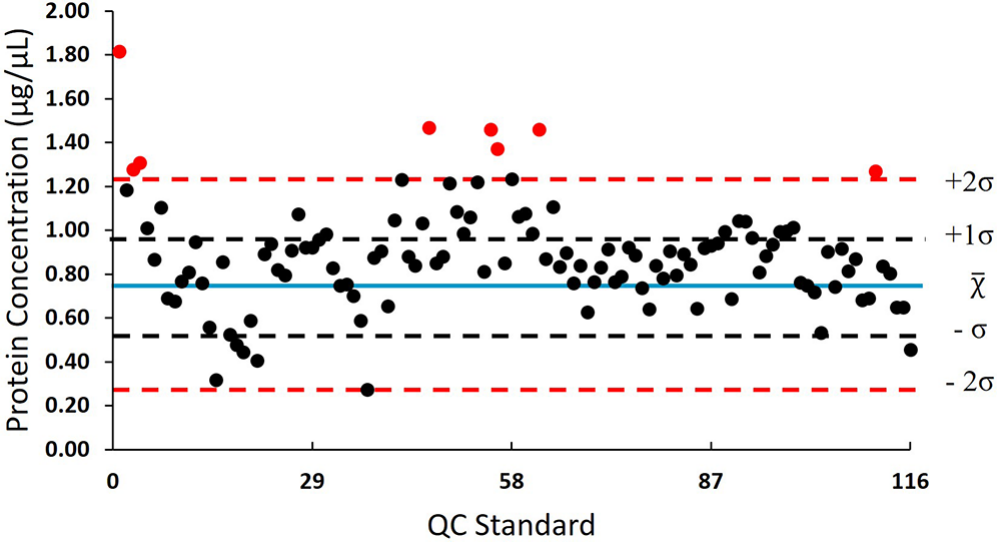
Protein concentrations of depleted human plasma standard protein (QC_std_) samples (*N* = 116) obtained from the BCA assay. The blue line represents the average (χ̅) concentration of 0.88 μg/μL, black lines represent one standard deviation (±σ) at 0.64 and 1.11 μg/μL, and dashed red lines represent two standard deviations (±2σ) falling at 0.40 and 1.36 μg/μL. Data points in black are within specification, and those in red are out-of-specification.

The QC specifications of CV were arbitrarily set to ≤10% between QC_std_ replicates, and for the BSA, standard curves were ≤5% based on prior analyses.^46^ Overall, BCA assays of depleted plasma showed a 5.52% CV, and many samples fell within one SD (±σ) of the 0.88 μg/μL mean (*x̅*) concentration (Figure 2). QC_std_ samples that fell outside the ±2σ window would be considered to be OOS; however, samples were not ready to be used in this study and were combined into a bulk sample since these were aliquots of the same plasma standard. It is recommended to reassay OOS QC_std_ samples to eliminate the need to combine the individual aliquots to accommodate this variation. Next, all QC_std_ samples (*N* = 116) were combined and generated ∼50 mg of surplus QC_std_ material for the remainder of the workflow and future projects.

Depletion efficiencies (DE), also described as column recovery by others,^45^ were measured daily by comparing the mass (μg) of protein injected onto the MARS-14 column and the mass of depleted protein using eqs 1–3. In summary, the volume of plasma standard injected onto the MARS-14 column (Inj_vol_) is calculated with eq 1
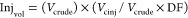
1where *V*_crude_ is the volume of the crude human plasma standard (40 μL), *V*_cinj_ is the diluted volume (in μL) of plasma standard injected onto the column, and DF is the dilution factor (i.e., ×4) used to dilute the crude sample. The amount of protein injected onto the column (*M*_crude_) was calculated with a crude BCA assay and eq 2
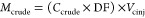
2where *C*_crude_ is the concentration of the crude sample. After depletion, another BCA assay determined the amount of low-abundance proteins (LAPs) (*M*_laps_) in the QC_std_. Finally, DE was calculated with eq 3
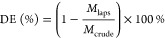
3

For protein depletion, it has been reported that MARS-14 columns do not efficiently capture targeted proteins.^27^ Therefore, a 90% MARS-14 DE was chosen as the desired specification because it is dependent on protein concentrations obtained from the BCA assay and not target identification that would require an LC–MS/MS check. If the results were OOS, participant plasma depletions were paused, and another QC_std_ aliquot was depleted for confirmation. If the subsequent efficiency met the specification, participant plasma sample depletion was resumed. If the subsequent efficiency was the OOS, the MARS-14 column was terminated, and a new column was introduced.

**Figure 3:**
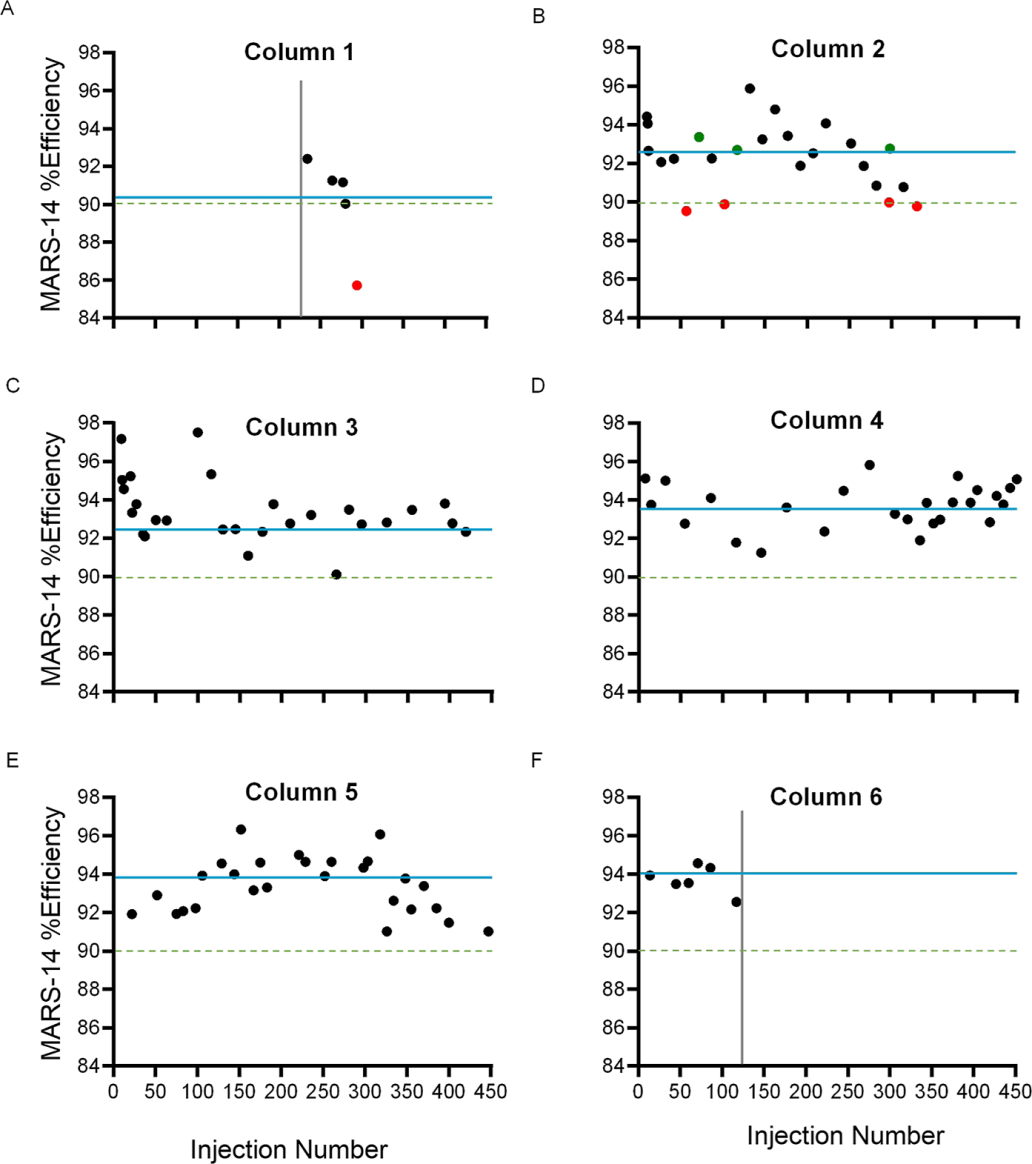
Calculated depletion efficiency percentages (Efficiency_std_) of QC_std_ across six multiple affinity removal system (MARS-14) columns (A–F). The blue lines represent average (χ̅) Efficiency_std_, while the green dotted lines represent the QC specification of 90% efficiency that must be met. Black points represent QC_std_ efficiencies that fell within the specification, and red points represent those that fell OOS. A subsequent injection of QC_std_ is measured to either confirm an OOS result or support the health status of the column unless the column lifetime was terminated by the operator. Green points show instances of efficiencies that fell within specifications following an OOS result.

A total of six MARS-14 columns were used (Figure 3), including Column 1 (Figure 3A) that was used in a previous plasma depletion study. It was the operator’s responsibility to check DE prior to continuing sample depletions. Columns were retired after two consecutive OOS DE results. Column injection lifetimes can exceed manufacturer recommendations and were maximized at 450 injections overtime (Figure 3C–E). It is noted that this recommended specification exceeds manufacturer recommendations; however, it is more cost-effective for larger studies. At the end of this study, operators were equipped with a column (Figure 3F) that could be used in future plasma depletion studies.

Further recommendations include the use of multiple instruments to complete large-scale depletions in less time. In this study, two HPLC systems were used, and QC_std_ retention times were highly consistent as the LAP fraction as within ±0.22 min of the mean (Figure S1).

### Digestion Efficiency

**Figure 4:**
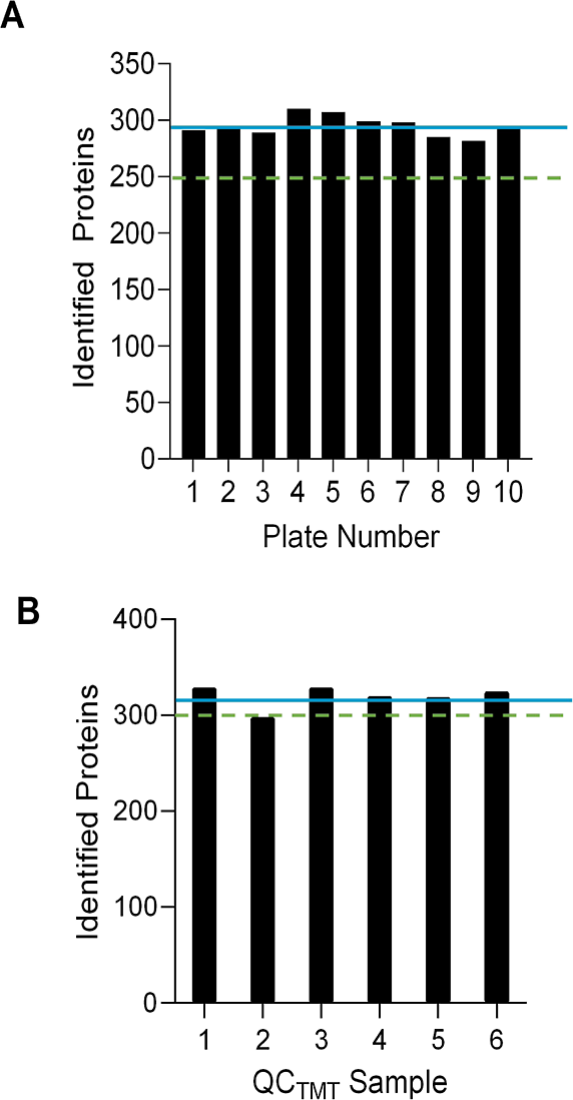
QC specifications for the number of identified proteins in automated digestion and TMT labeling. Green dotted lines represent the QC specifications that were met before cohort samples continued to the next step in the workflow. (A) Identified proteins of 96-well plates (*N* = 10) required for automated protein digestion. The QC specification of ≥250 proteins was met with an average (χ̅ blue line) of 294 proteins. (B) Identified proteins of six QC_TMT_ sample batches created from 96-well plates (*N* = 12) used in automated TMT labeling. QC_TMT_ batches 1 and 3–6 met the QC specification of ≥300 proteins. QC_TMT_ batch 2 fell short of the QC specification with 298 proteins. The overall average (χ̅ blue line) was 320 identified proteins.

Tryptic digestion was performed using a robotic liquid handler and required ten 96-well plates (Figure 1) to accommodate both QC samples and cohort sample batches. Digestion and desalting spanned two days. Six of the 12 QC_dig_ samples from each plate were analyzed with an LC–MS/MS check. RAW files (*N* = 60) were evaluated with PD to obtain the number of identified proteins across the ten plates. All plates had an average of 294 proteins with a 4.80% CV (Figure 4A). While prior data (not shown) suggested a minimum level of 250, future studies would be adjusted, as this study suggests that a minimum of 275 identified proteins is achievable for each plate using the LC–MS/MS conditions described (see Materials and Methods). We note that each laboratory would have to determine this metric based on their instrumental performance and LC conditions.

Digestion efficiency was also evaluated by the number of tryptic miscleavages. Although cleavage precision at lysine residues is enhanced with the use of a trypsin/Lys-C mix, simultaneously considering one or more missed cleavages for each peptide can improve quantification.^47^ The QC_dig_ samples in this study produced an average of 2956 peptides, with 87% having one site of miscleavage (Figure S2). Identified protein abundances, number of peptides, and PSMs, along with FDR confidences for each check, are provided (Table S1).

### Labeling Efficiency

TMT labeling was performed in an automated fashion and required a total of twelve 96-well plates (Figure 1). The *S*_pool_ was labeled with TMT_zero_ and reserved for an instrument QC for final LC–MS/MS analysis.^41^ The remaining six QC_dig_ samples were labeled with TMTpro and produced six batches of TMTpro across two plates (Figure 4B). These samples were combined after labeling to make the six corresponding QC_TMT_ samples. LC–MS/MS analysis of QC_TMT_ resulted in an average of 320 proteins with a 3.31% CV. Comparing QC_TMT_ to QC_dig_, protein counts were higher, likely due to the use of an Orbitrap Fusion Lumos Tribrid system for QC_TMT_ checks, which has higher sensitivity than the Q-Exactive system.

The LE of the QC_TMT_ samples (given as a percentage) was calculated with the number of PSMs (eq 4)
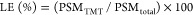
4where PSM_TMT_ is the number of PSMs with a TMT modification and PSM_total_ is the total number of PSMs identified.

**Figure 5:**
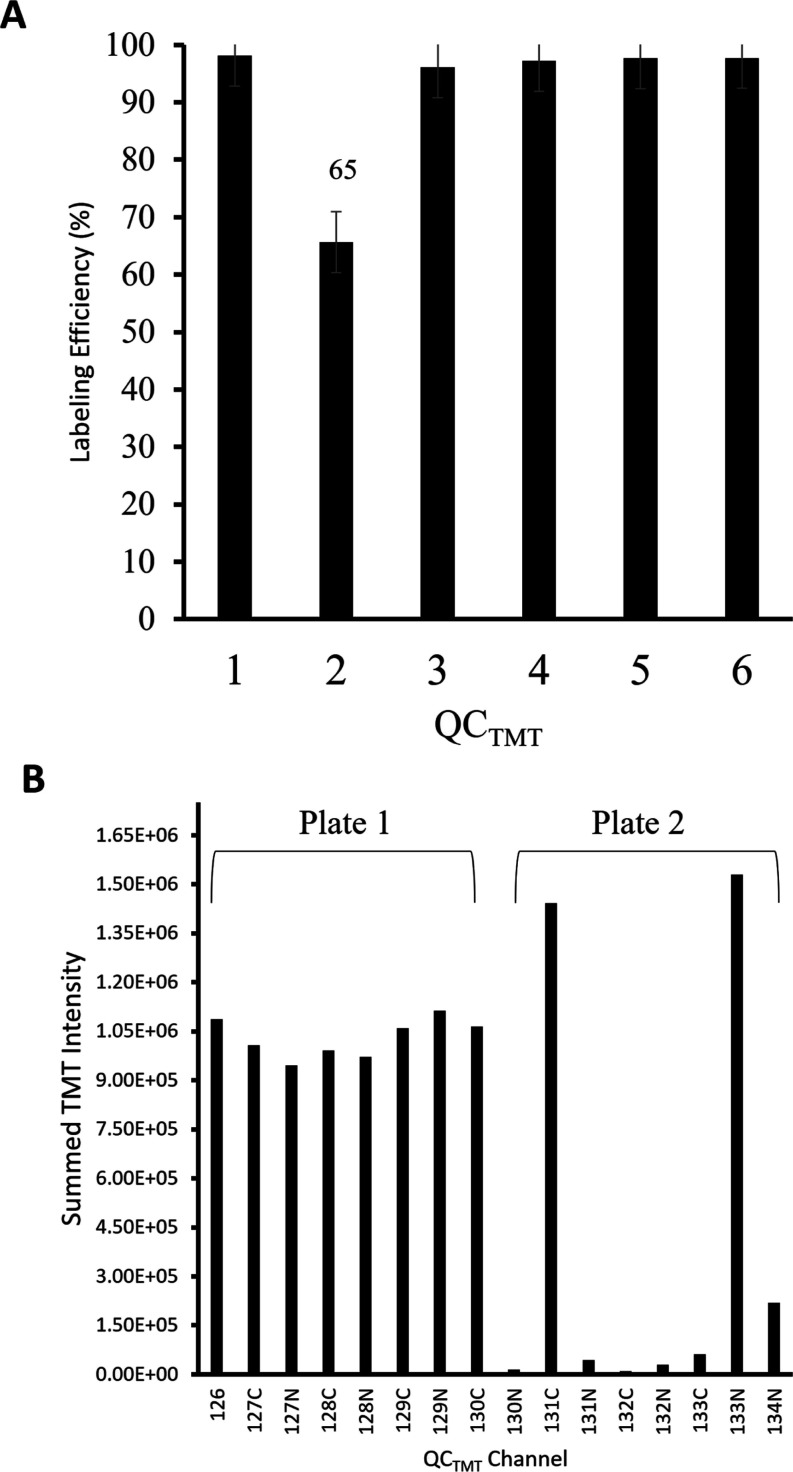
Labeling efficiency (Efficiency_TMT_) checks and investigation of automated TMT labeling. (A) Calculated Efficiency_TMT_ for six QC_TMT_ sample batches. All batches met the QC specification of ≥∼98% efficiency (red line), except batch 2 at only 65%. (B) QC_TMT_ batch 2 peptide abundances. Channels 126–130N represented automated labeling plate 1, while 130C–134N was plate 2. Plate 2 abundances were inconsistent and prompted operators to evaluate automated labeling processing and refrain from using QC_pool_ wells for final LC–MS/MS analysis from this plate.

QC_TMT_ batches 1 and 3–6 met a 98% LE consistent with manufacturer expectations.^44,48^ QC_TMT_ batch 2 fell into the OOS with a LE of 65% (Figure 5A). Figure 4B also reveals an OOS result of 298 identified proteins for QC_TMT_ batch 2. Due to this batch representing both plates 3 and 4, peptide abundance analysis was conducted to help identify which plate may have caused the low efficiency (Figure 5B). TMTpro channels 130C–134N, corresponding to plate 4, revealed summed TMT intensities that were less than the *x̅* signal of 1.1 × 10^6^, taken across all other reporter ion channels from the three preceding plates. Operators believe this was a result of insufficient solvent availability when the robotic liquid handler reached plate 4. This was noticed in real-time and was replenished before the method continued for the remaining plates; however, these four QC_pool_ samples were deemed unusable for the final LC–MS/MS analysis. The other samples on plate 4 will be assessed for variability upon completion of data acquisition elsewhere. Overall, the six QC_TMT_ batches produced an average of 320 proteins, 8134 PSMs, a 92% average LE, and a 3.30% CV. Identified protein abundances by channel, number of peptides, and PSMs, along with FDR confidences for each QC_TMT_ batch, are provided (Table S2).

### Fractionation Reproducibility

BSA was digested (QC_BSA_, *N* = 40) and used to track the daily HPLC performance during RP fractionation. Our analysis consistently produced 51 BSA peaks (Figure S3). Chromatograms were divided into four quadrants (*Q*), and two peaks from each were chosen to track the t_R_ profiles. Peaks were chosen based on abundance and consistent detection in respective quadrants after 3 days of initial sample injections (Figure S3). Minimum and maximum times were established as acceptable ranges to serve as the QC specifications for the tracked peaks. Retention time reproducibility in each quadrant was high (Table 3), with the first peak in Q1 (a solvent peak) showing the greatest variance at 8.89% CV. We note that sample peaks 2–7 had an average CV of 6.5% for analyte *t*_R_ values.

**Table 3:** Peak Analysis of QC_BSA_ Fractionation Peaks^a^

RT (min)	AVG^b^	SD	% CV	min^b^	max^b^
peak 1 (solvent)	7.73	0.69	8.89	7.77	8.27
peak 2	14.67	0.36	2.48	13.64	14.14
peak 3	22.30	0.93	4.19	21.65	22.15
peak 4	29.21	0.26	0.88	28.78	29.28
peak 5	34.42	0.24	0.69	34.28	34.78
peak 6	39.99	0.94	2.36	40.35	40.85
peak 7	46.38	3.36	7.25	47.68	48.18
peak 8 (solvent)	54.05	0.02	0.03	54.04	54.08

a Measurements were taken at the maximum peak height.

b Digested bovine serum albumin (*N* = 2) denoted as QCBSA; abbreviations: AVG, average; BSA, bovine serum albumin; CV, coefficient of variance; SD, standard deviation.

It should be noted that some retention shifts may arise due to inadequate column performance or expired lifetime, the use of independent preparations of QC_BSA_ digests, or the pH instability of buffers. Guard columns were installed to minimize maintenance issues and changed periodically. This helped reduce flushing times and instrument downtime. It will be helpful to estimate the number of days required for fractionation to ensure that enough QC_BSA_ is generated from a single preparation. A large stock of QC_BSA_ can also be generated using automated digestion and is reserved for future studies.

### Designing Future QC Metrics

All QC metrics were designed to represent the complexity of the automated plasma proteomic workflow and were influenced by operator laboratory training and established protocols. Based on the QC samples analyzed in this study, we have recommendations for acceptable specifications for each step of the workflow. For depletion, 93% DE was an acceptable specification for the MARS-14 column and requires consistent maintenance. Operators worked well with shared sample tracking spreadsheets and learned consistency with operator task assignments, which also helped to reduce variation. In automated digestion, the ≥275 protein identification specification would be determined based on the LC–MS/MS acquisition methods, and after some minimal number (we recommend three) of QC samples are analyzed. As sample preparation workflows differ from project to project, this number should be determined by a consistent history of digestion and LC–MS/MS analysis.

There is an opportunity to optimize the LC–MS/MS digestion checks for simplified and faster analyses. However, specifications for TMT labeling exceeded and trended well with previously reported multiplexing experiments.^22,39,40^ LC–MS/MS data acquisition methods used are recommended to more closely resemble final analysis methods^32^ for the overall cohort study. For fractionation, BSA can be added to automated digestion plate layouts to decrease the operator workload. Lastly, different fractionation QC sample types^49^ can be used instead of digested BSA.

Increased use of automation in proteomic sample preparations requires that QC metrics are embedded and measure both experiment efficiency and robotic liquid handler performance. These metrics can be differentiated by internal and external QC sample types. Internal QC samples (QC_batch_) may be defined as samples within initial batches that serve as TMT QC channels for normalization in final LC–MS/MS analyses.^39,40^ This study utilized two TMTpro channels for internal QCs and will be presented in a future study upon completion of the data acquisition. External QCs may be defined as samples outside of a sample batch that represent the assay itself. An example would be the QC_dig_ samples that are removed for digestion checks. External QCs can also be used for instrument monitoring, and in this study, they were tagged with TMTzero. All of these approaches for QC implementation in sample preparation steps can be adjusted and optimized for translation to other sample preparation strategies and LC–MS/MS data acquisition methods.

### Study Strengths and Limitations

The major strengths of this work are found in the study design. Semiautomation of BCA assays, digestion, desalting, and TMT labeling significantly reduced user error and saved time. The generation of the five QC sample types allowed operators to measure variation without utilizing patient samples. These QC samples were generated in excess for use in future plasma sample preparations, with the QC_pool_ sample particularly useful for comparing across instruments and cohort studies. However, the high-throughput nature of this study presents some limitations. Very involved planning was required to store, organize, and process large numbers of plasma samples. The use of automation required designing 96-well plates that organized sample batches and included internal and external QCs and the random generation of sample placement. It also required a calculation of the additional amounts of reagents necessary for liquid handler reservoirs. Additionally, much consideration was given to instrument availability, run times, supply ordering, and operator training and schedules. These considerations were managed through the creation of standard operating procedures (SOPs) and data tracking templates. Their use made it easy for operators to track samples and manage tasks at each step of the workflow.

Time is a major limitation of this work. The overall workflow is intensive, with four main steps, each with additional analyses and efficiency checks. To summarize, the sample preparation workflow, including QC testing for the patient samples, occurred over ∼22 months. This time includes operator onboarding and training, operator transitions, instrument PMs, method optimization, etc. Inter- and intraday measuring of QC metrics extended sample processing times with the trade-off of quality data. Depletion and fractionation required consistent use of HPLC systems over three months for each workflow step. LC–MS/MS gradients for large cohort studies should be balanced to allow for adequate separation of complex mixtures, however, with overall regard to project throughput. Finally, the QC measures reported here were specific to the bottom-up proteomics strategy utilized herein and would require adjustment and tailoring for other proteomics sample preparation strategies.

## Conclusions

The establishment of QC metrics for large-scale and automated sample preparation workflows can help manage the experimental variability of high-throughput LC–MS/MS plasma proteomic studies. Although the created QC sample types and specifications are only examples, we have demonstrated the capabilities of several QC metrics across multiple sample preparation steps and with <10% CV. The QC specifications generated prior to large-scale LC–MS/MS analysis studies inform of irregularities in sample preparation that can guide efficient troubleshooting. This study was performed in the context of preparing a large cohort of patient plasma samples for LC–MS/MS analyses using a multistep and automated sample preparation workflow. QC metrics should be adjusted based on desired preparation steps, sample types, and cohort sizes. The standardization of representative and well-integrated QC metrics can ultimately increase the confidence of large-scale plasma sample preparations and is a growing and necessary area for proteomics researchers to address.

## Supporting Information Available

Summary of protein depletion across two HPLC systems; summary of digestion QC check across ten 96-well plates; and chromatogram of QC_BSA_ reversed-phase fractionation (PDF)
Protein, peptide, and peptide spectral match data for QC_dig_ plates (XLSX)
Protein, peptide, and peptide spectral match data for QC_TMT_ plates, including TMT reporter ion intensities (XLSX)
