# Supplementary material for: Establishing Quality Control Metrics for Large-Scale Plasma Proteomic Sample Preparation
Source: ACS Meas Sci Au. 2024 Apr 29;4(4):442–51. doi: 10.1021/acsmeasuresciau.3c00070 (PMC11342454; doi:10.1021/acsmeasuresciau.3c00070)
Supplement: Supplementary file 1 — tg3c00070_si_001.pdf [file tg3c00070_si_001.pdf]

Supporting Information

Establishing Quality Control Metrics for Large-Scale Plasma Proteomic Sample Preparation

Nekesa C. Oliver[1], Min Ji Choi[1], Albert B. Arul[1], Marsalas D. Whitaker[1], and Renã A. S. Robinson[1-5*]

[1] *Department of Chemistry, Vanderbilt University, Nashville, TN 37235, United States*
[2] *Vanderbilt Memory and Alzheimer's Center, Vanderbilt University Medical Center, Nashville, TN 37212, United States*
[3] *Vanderbilt Institute of Chemical Biology, Vanderbilt University, Nashville, TN 37232, United States*
[4] *Vanderbilt Brain Institute, Vanderbilt University, Nashville, TN 37232, United States*
[5] *Department of Neurology, Vanderbilt University Medical Center, Nashville, TN 37232, United States*

[*]Author to whom correspondence should be addressed:

Prof. Renã A. S. Robinson

Department of Chemistry

Vanderbilt University

5423 Stevenson Center

Nashville, TN 37235

Fax: 615-343-1234

Tel: 1-615-343-0129

Email: rena.as.robinson@vanderbilt.edu

Running Title: Quality control for plasma proteomics sample preparation

TABLES

Supplemental Table S1. Protein, peptide and peptide spectral match data for QC_{dig} plates.

Supplemental Table S2. Protein, peptide, peptide spectral match, and reporter ion intensity data for QC_{TMT} for each batch.

FIGURES

Figure S1. A summary of protein depletion across two HPLC systems. QC_{std} depletions produced reproducible peaks over a 38-min gradient, six MARS-14 columns, and 86 days. HPLC 1 (B) was the main instrument and had 79 injections while HPLC 2 (A) had 37 injections. While all unbound peaks had negligible variation in retention time (t_R), a combined profile of bound and solvent peaks (C) reveals a slight t_R difference between HPLC 1 and 2. There were 116 total QC_{std} injections.

Figure S2. A summary of the digestion QC check across ten 96-well plates. The number of identified peptides is an average of six wells across an individual plate. An average of 87% of proteins were found having no mis cleavages.

Figure S3. Chromatogram of QC_{BSA} reversed-phase fractionation. A 60-min gradient was divided into four 15-min quadrants (Q1 - Q4) from which two peaks were selected for retention time (t_R) peak analysis.

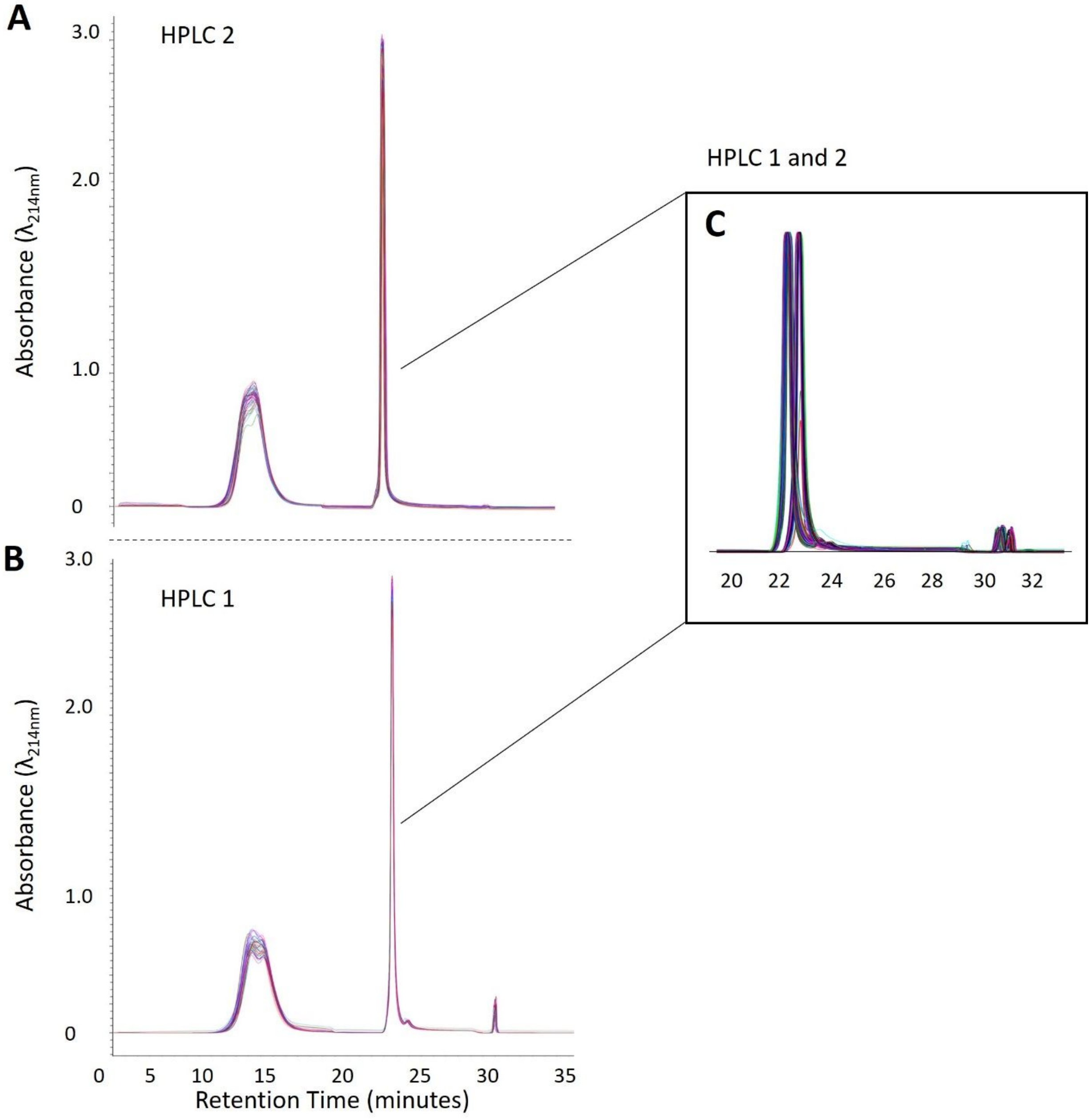
A
HPLC 2
Absorbance (λ_{214nm})
3.0
2.0
1.0
0
B
HPLC 1
Absorbance (λ_{214nm})
3.0
2.0
1.0
0
0 5 10 15 20 25 30 35
Retention Time (minutes)
HPLC 1 and 2
C
20 22 24 26 28 30 32

Figure S1

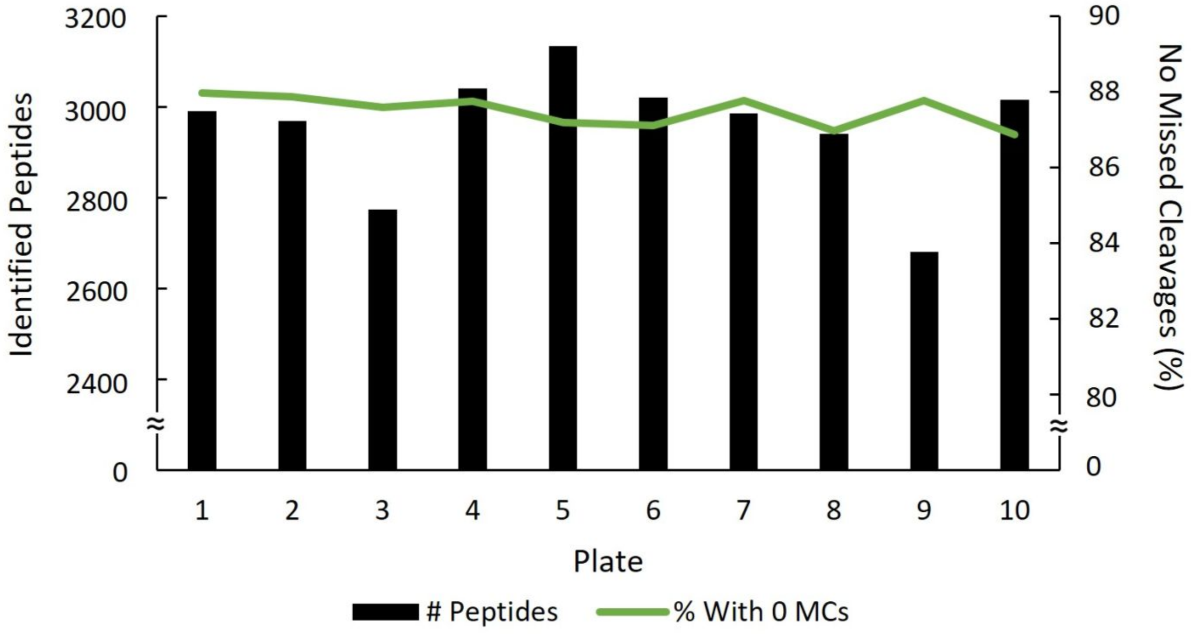
3200
3000
2800
2600
2400
0
Identified Peptides
90
88
86
84
82
80
0
No Missed Cleavages (%)
1
2
3
4
5
6
7
8
9
10
Plate
Peptides
% With 0 MCs

Figure S2

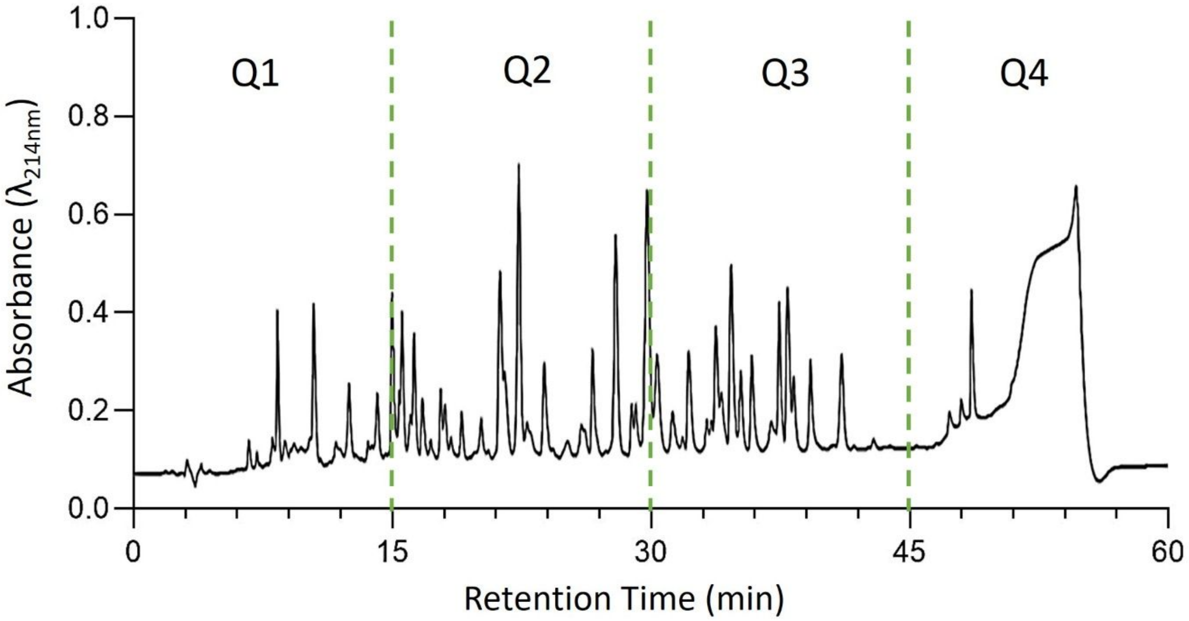

Q1
Q2
Q3
Q4
Absorbance (λ214nm)
1.0
0.8
0.6
0.4
0.2
0.0
0
15
30
45
60
Retention Time (min)

Figure S3
